# Comment on "Association between lipoprotein(a) and coronary heart disease risk in type 2 diabetes mellitus and evaluation of statin treatment effects"

**DOI:** 10.1590/1806-9282.20250801

**Published:** 2025-10-27

**Authors:** Koray Kalenderoglu, Mert Ilker Hayiroglu, Tufan Cinar

**Affiliations:** 1 Health Sciences University, Dr. Siyami Ersek Thoracic and Cardiovascular Surgery Center, Department of Cardiology – Istanbul, Turkey.; 2 University of Maryland Midtown Campus, Department of Medicine – Baltimore (MD), United States.

Dear Editor,

We recently read with great interest the article titled "Association between lipoprotein(a) and coronary heart disease risk in type 2 diabetes mellitus and evaluation of statin treatment effects" by Li Li and Liwu Xu, published in your esteemed journal^1^. The authors deserve commendation for tackling a crucial clinical subject: the significance of lipoprotein(a) [Lp(a)] as a residual risk factor for coronary heart disease (CHD) in patients with type 2 diabetes mellitus (T2DM).

The study presents several notable strengths. First, it sheds light on an emerging and clinically relevant topic, as Lp(a) is increasingly recognized as an independent predictor of cardiovascular risk. The details around patient selection and categorization based on coronary angiography were clearly articulated, and the statistical analyses, including logistic regression and receiver operating characteristic (ROC) curve evaluations, were well-executed and comprehensive. Moreover, the exploration of statin therapy's effects on lipid profiles, particularly the finding that Lp(a) levels were not significantly decreased, adds practical insight into lipid management for high-risk populations.

However, there are limitations that should be addressed. The relatively small size of the single-center, retrospective cohort may impact the applicability of the findings beyond the study population. Additionally, the absence of information on glycemic control parameters, such as HbA1c, hinders a thorough assessment of cardiovascular risk. Although the authors identified a diagnostic threshold for Lp(a) at 97.5 mg/L, this cutoff should be approached with caution, given the sample size, and should not be generalized for clinical practice until external validation has been obtained.

The authors prescribed rosuvastatin 10 mg to all participants, but the study falls short in explaining the reasoning behind choosing rosuvastatin over other statins or the justification for the low dosage of 10 mg. Current guidelines recommend that the target low-density lipoprotein-cholesterol (LDL-C) level for both acute and chronic coronary syndrome patients be <1.4 mmol/L (55 mg/dL) and a ≥50% reduction in LDL-C from baseline. While this threshold is also applicable to very high-risk T2DM patients, it suggests that those patients should achieve an LDL-C target of <1.8 mmol/L (<70 mg/dL) along with a >50% reduction from baseline. Furthermore, it is preferable to prescribe a high-intensity statin at the maximum tolerated dose to reach LDL-C targets^2,3^. Considering that different patient populations may have varying LDL targets, the authors should note the limitation that patients were followed on a fixed and low-dose statin for 6 months without consideration for specific LDL target values and without differentiating between patients with CHD and those with T2DM.

In summary, despite these limitations, this study offers valuable contributions by emphasizing the importance of early detection and management of elevated Lp(a) levels in T2DM patients. I encourage the authors to pursue prospective, multicenter studies with larger cohorts for further validation of these findings and to investigate therapeutic strategies aimed at reducing Lp(a) levels.

## Data Availability

The datasets generated and/or analyzed during the current study are available from the corresponding author upon reasonable request.
